# Advancement of Metatranscriptomics towards Productive Agriculture and Sustainable Environment: A Review

**DOI:** 10.3390/ijms23073737

**Published:** 2022-03-29

**Authors:** Siti Suhailah Sharuddin, Norhayati Ramli, Mohd Zulkhairi Mohd Yusoff, Nor Azlan Nor Muhammad, Li Sim Ho, Toshinari Maeda

**Affiliations:** 1 Department of Bioprocess Technology, Faculty of Biotechnology and Biomolecular Sciences, Universiti Putra Malaysia, Serdang 43400, Selangor, Malaysia; suhaila.sharuddin@yahoo.com (S.S.S.); mzulkhairi@upm.edu.my (M.Z.M.Y.); 2 Laboratory of Biopolymer and Derivatives, Institute of Tropical Forestry and Forest Products (INTROP), Universiti Putra Malaysia, Serdang 43400, Selangor, Malaysia; 3 Institute of Systems Biology (INBIOSIS), Universiti Kebangsaan Malaysia, Bangi 43600, Selangor, Malaysia; norazlannm@ukm.edu.my; 4 Sime Darby Plantation Technology Centre Sdn Bhd, Sime Darby Plantation, Serdang 43400, Selangor, Malaysia; ho.li.sim@simedarbyplantation.com; 5 Department of Biological Functions Engineering, Graduate School of Life Science and Systems Engineering, Kyushu Institute of Technology, 2-4 Hibikino, Wakamatsu, Kitakyushu 808-0196, Japan; toshi.maeda@life.kyutech.ac.jp

**Keywords:** metatranscriptomics, pollution, environmental sustainability, productive agriculture, bioindicator

## Abstract

While chemical fertilisers and pesticides indeed enhance agricultural productivity, their excessive usage has been detrimental to environmental health. In addressing this matter, the use of environmental microbiomes has been greatly favoured as a ‘greener’ alternative to these inorganic chemicals’ application. Challenged by a significant proportion of unidentified microbiomes with unknown ecological functions, advanced high throughput metatranscriptomics is prudent to overcome the technological limitations in unfolding the previously undiscovered functional profiles of the beneficial microbiomes. Under this context, this review begins by summarising (1) the evolution of next-generation sequencing and metatranscriptomics in leveraging the microbiome transcriptome profiles through whole gene expression profiling. Next, the current environmental metatranscriptomics studies are reviewed, with the discussion centred on (2) the emerging application of the beneficial microbiomes in developing fertile soils and (3) the development of disease-suppressive soils as greener alternatives against biotic stress. As sustainable agriculture focuses not only on crop productivity but also long-term environmental sustainability, the second half of the review highlights the metatranscriptomics’ contribution in (4) revolutionising the pollution monitoring systems via specific bioindicators. Overall, growing knowledge on the complex microbiome functional profiles is imperative to unlock the unlimited potential of agricultural microbiome-based practices, which we believe hold the key to productive agriculture and sustainable environment.

## 1. Introduction

**Figure 1:**
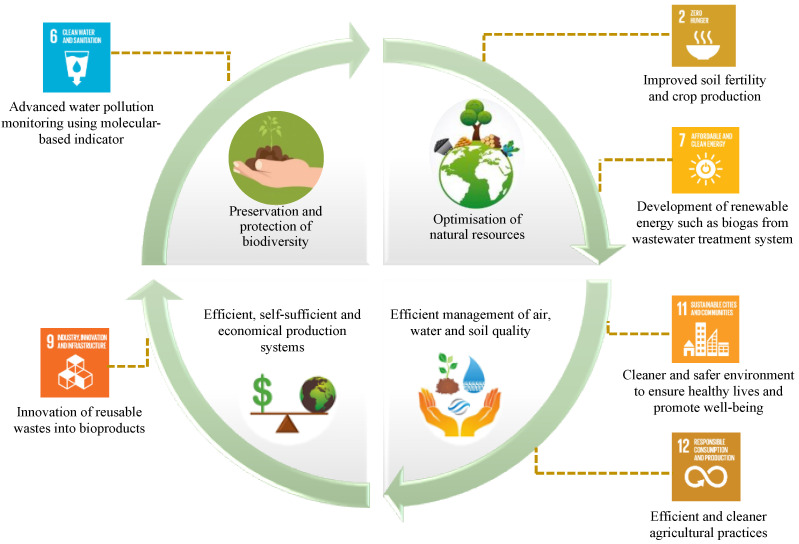
Advancing the key principles of sustainable industrial agriculture and related Sustainable Development Goals (SDGs) through microbiome-based innovations.

With the global population projected to reach 9 billion by 2050, farm productivity is also required to be increased up to 70–100% ideally to meet food, fuel, and fibre demands [1]. For that, excessive usage of chemical fertilisers and pesticides has been unavoidable to improve agricultural productivity [2], which unfortunately has caused environmental pollution, soil infertility, and the loss of biodiversity, affecting the overall ecosystem sustainability and potentially being hazardous to human health [3]. Therefore, as the current agricultural practices have become increasingly unsustainable, there is an essential need to rectify this issue. Globally, the Sustainable Development Goals (SDGs) formulated by the United Nations in 2015 have been widely promoted to ensure development sustainability, with the 17 SDGs forming the backbone of this plan. The SDGs are adopted by factoring in the relationship of the key ecological processes and relevant human behavioural activities, which are highly interdependent [4]. It is well established that ecological processes are primarily mediated and regulated by microbiomes, the predominant form of life on the planet. Overtaking the advantage of the existing synergistic microorganisms in regulating the biogeochemical cycle in the environment [5], a subtle way of exploiting the beneficial microbes and the carried essential genes involved in certain processes could be embarked upon in order to resolve the negative impacts of aggressive agricultural activity and potentially advance the related SDGs such as SDG 2 (Zero Hunger), SDG 6 (Clean Water and Sanitation), SDG 7 (Affordable and Clean Energy), SDG 9 (Industry, Innovation, and Infrastructure), SDG 11 (Sustainable Cities and Communities) and SDG 12 (Responsible Consumption and Production) through microbiome-based innovations (Figure 1).

With this motion, researchers are steering the application of next-generation sequencing (NGS) towards achieving a comprehensive understanding of the role and potential of beneficial microbes in sustainable agricultural practices where productivity and profit are maximised, environmental damage is minimised, and natural resources are preserved. NGS started about two decades ago with a microbial taxonomic diversity analysis using amplicon sequencing (16S/18S rRNA sequences) [6,7]. Amplicon sequencing is mainly used to characterise the diversity and structural compositions of microbial communities, and many studies have successfully employed this approach to explore the microbial taxonomies and phylogeny in different environments, such as surface water [8], agriculture wastewater treatment ponds [9], and activated sludge reactors [10]. Amplicon-based gene sequencing gained immense popularity due to its low cost, simple sample preparation protocols, and wide accessibility of bioinformatics tools [11]. While gene amplicon sequencing provides unparalleled insight into the microbiome’s nature, this technique can only provide profiling of community structures without having an accurate overview of their functionality [12].

As technology advances, researchers have started to work with whole-genome shotgun (WGS) metagenomics, contributing to the discovery of both the taxonomic and functional diversity of a particular community [13,14,15]. Shotgun metagenomic sequencing is a powerful technique to infer the taxonomical structure by sequencing all genomic DNA fragments in a community while avoiding the biases observed in amplicon sequencing due to the non-requirement of amplification before sequencing [11]. However, as WGS is a type of descriptive-based analysis, it can only provide a predictive functional profile [16]. Aside from that, shotgun metagenomics is also unable to differentiate the active from the inactive members of the microbiome [17]. Consequently, it cannot address a part of the research question relating to which community contributes to the observed ecosystem activity and which are merely present in a dormant state [6]. Therefore, the demand for a more comprehensive approach to infer the microbiome’s functional profiles brought forth the emergence of metatranscriptomics.

**Figure 2:**
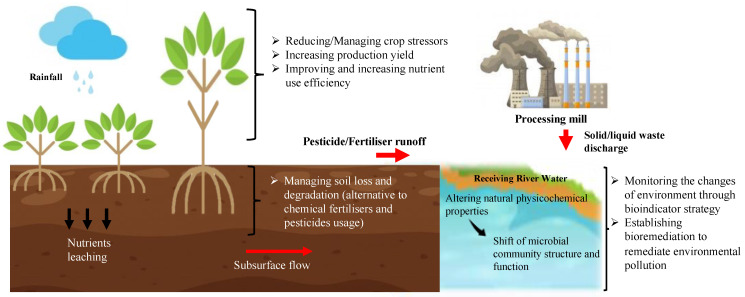
Schematic presentation of the major goals in promoting environmentally sustainable agriculture.

Metatranscriptomics is defined as the assessment of a gene’s expression in a population or a whole community [16]. Metatranscriptomics is used to expand our knowledge of the microbial community’s functions in terms of gene expression, regulations, and pathways, which significantly correspond to environmental changes. In the last decade, various studies have employed large-scale metatranscriptomics analyses for complex environmental samples such as the study of soil fertility [18], biofertilisers [19], crop disease [20], and second-generation biofuel [21]. As metatranscriptomics applications are gaining more attention, it is important to understand how we can utilise the knowledge on microbial genes and functionality to achieve a sustainable environment (Figure 2). Thus, the current applications of environmental metatranscriptomics in microbiome-based innovations and its possible contribution towards realising environmentally sustainable agriculture by harnessing the microbiome’s potential in developing healthy soil and pollution bioindicators will be further discussed in this review.

## 2. Improving Agricultural Soil Quality without Excessive Chemical Fertiliser Input

**Figure 3:**
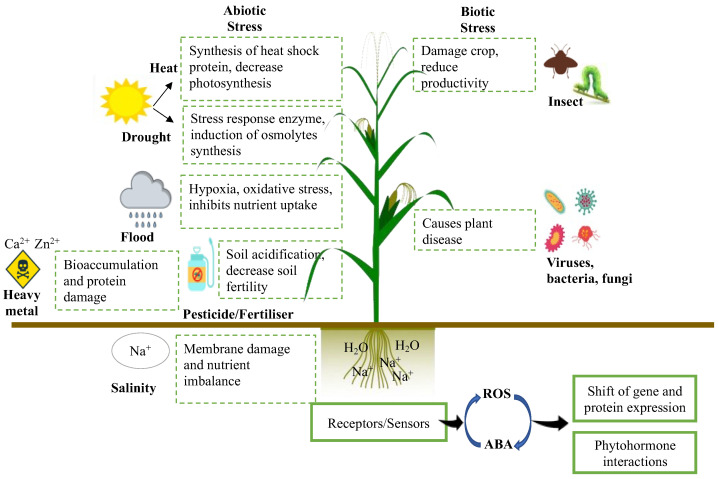
The effects of abiotic and biotic stresses on crop productivity.

The ideal soil quality in agriculture is characterised by the ability to suppress a wide range of crop diseases and provide the optimum nutrients needed for crop growth and productivity, which are fundamental for crops experiencing various abiotic and biotic stresses (Figure 3). The most typical means to maintain and even improve the said soil attributes would be the application of chemical fertilisers, which would assist in providing different necessary nutrients such as potassium, phosphorus, and nitrogen to crops, enabling greater growth and productivity [2]. Nevertheless, the continuous excessive use of chemical fertilisers results in alteration of the optimum soil pH and heavy metal accumulation in agricultural soils, consequently leading to soil infertility and decreased crop productivity [21]. Furthermore, excessive reliance on chemical fertilisers may also cause deleterious effects on the environment due to the release of damaging greenhouse gases into the atmosphere [22] and eutrophication of the waterways [23,24].

For many decades, small farmers have been using organic materials such as livestock manure in composting as an alternative to inorganic fertilisers in supplying the nutrients needed for crop production, further sanctioning the organic farming concept [22,23]. Livestock manure is an excellent source of essential nutrients for plants to grow, such as nitrogen, phosphorus, and potassium. Aside from animal manure, other organic wastes such as agricultural biomass and food wastes can also be treated and converted into organic fertilisers through the composting process. Despite that, there is an issue relating to the consistency of the compost quality for every production batch and the effectiveness of the compost [24], causing its application to be infeasible for widespread adoption in the agriculture industry. In addition, the soil amendment strategy using biochar has also been widely reported as a promising approach to treat infertile soil for decades [25,26,27], but the efficacy of this strategy is not well established. Hence, as the application of compost and biochar presents a promising alternative to chemical fertilisers, efforts are directed at elucidating the functional profiles of the beneficial microbiome in various environments (Table 1), which can be used to improve both strategies for extensive usage in agriculture.

On top of that, there is a growing research area for exploiting rhizospheric beneficial microbes, namely plant growth-promoting rhizobacteria (PGPR), to serve as biofertilisers and nutrient uptake enhancers for plants [35]. The PGPRs are the bacteria that colonise the plant roots or rhizosphere and directly promote plant growth by immobilising nutrients or acting as a defence regulator [36]. However, the lack of data on the lower taxonomic levels and the underlying mechanisms raises questions on the relevance and feasibility of the proposed PGPR strains to be globally applied in every climatic situation [37]. Luckily, with the advancement of metatranscriptomics, it is now possible for us to further understand and exploit the beneficial microbes, purposively adding value to the compost and biochar applications while simultaneously reducing the dependency on chemical inputs, thereby contributing directly to SDGs 2, 9, and 12.

For instance, the application of metatranscriptomics to investigate the gene expression of PGPRs in Mexican maize was conducted to comprehend the bacteria–bacteria interactions in roots [38]. By focussing on the gene expression of *Rhizobium phaseoli* in the presence of other PGPRs (*Sinorhizobium Americanum*, *Azospirillum brasilense*, *Bacillus amyloliquefaciens*, and *Methylobacterium extorquens*), the authors discovered several significantly upregulated genes associated with nitrogen fixation proteins (NifX and NifZ), *the* nitrogenase cofactor biosynthesis protein (NifB)*,* and the nitrogenase stabilisation protein (NifW). Supported by several other reports, it was shown that the regulation of the *nif* gene machinery in the maize root is directly linked to the supply of ATP required to provide adequate energy for nitrogen fixation to occur [39,40]. A similar study was conducted on the sugarcane root microbiome to unveil plant growth–PGPR interaction [41]. From there, it can be comprehended that the essential enzyme activities such as peroxidase and superoxide dismutase were significantly enriched in the sugarcane roots after the inoculation of *Burkholderia anthina,* which led to growth promotion. Based on these studies, it is fair to deduce that PGPR strains can improve plant nutrient uptake through various mechanisms such as the association of nitrogen fixation, phosphate solubilisation, and hormone production. Hence, the exploration of the beneficial PGPR strains is proposed to aid in the improvement of agricultural microbiome-based practices.

Following this, there have been efforts to combine the discovered PGPR strains with compost as multipurpose biofertilisers that supply the necessary nutrients, improve crop tolerance towards stress, and maintain soil fertility [42,43]. One of the studies inoculated *Bradyrhizobium japonicum* and *Bacillus subtilis* as the PGPR, which added value to the compost with a proven ability to reduce the chemical fertilisers’ usage in the cultivation of *Brassica integrifolia*, *Brassica chinensis* L., and *Brassica juncea* L. by 50% [44]. The knowledge on PGPR mechanisms motivated the combination of PGPR *Pseudomonas* sp. with rock phosphate-enriched compost (poultry litter) for maize, whereby a prominent increment of phosphorus uptake was observed, which was explainable by the phosphorus-solubilising metabolisms exuded by this strain. Aside from the improvement in phosphorus uptake, the inoculation of *Pseudomonas* sp. also increased the chlorophyll content in the crop, which resulted in an incremental grain yield of the wheat crop [43]. Nevertheless, it is crucial to understand the PGPR mechanisms and metabolisms prior to actual application, as even though some strains are beneficial, a few of them are known as opportunistic pathogens, such as *Pseudomonas aeruginosa* [45]. With appropriate strategies and a comprehensive understanding of the PGPR mechanisms through metatranscriptomics, PGPR inoculation may further increase the efficiency of composts.

The functional potentials of various PGPR strains are also being utilised by combining them with biochar as a method to amend and thus improve the soil condition through the improvement of nutrient retention and availability, where biochar acts by enhancing the cation exchange capacity, surface area, and nutrient supply of the soil [46,47]. It is expected that PGPR inoculation with biochar will further improve the potency of biochar in improving soil conditions. Favouring the co-application of biochar and PGPR *Bacillus megaterium*, a successful experiment manifested improved soil fertility, with a significant increase in inorganic nitrogen due to the upregulated nitrogen fixation by the PGPR and reduced nitrogen leaching by the biochar. Additionally, the combination of PGPR and biochar significantly heightened the nitrate-nitrogen content and total potassium in the soil [48], which further justifies the practicality of this strategy in improving soil health.

It is well known that the excessive application of pesticides to soil and crops may contribute to hydrocarbon pollution in soil [49]. Hydrocarbon pollution may restrict natural processes such as plant transpiration, respiration, and the photosynthetic rates. For this reason, studies documenting the functional potential of PGPR strains in soil are actively conducted to determine their underlying mechanisms in pollutant degradation, which can be exploited to recover the hydrocarbon-polluted soil and improve soil fertility. Through transcriptional profiling using high-throughput metatranscriptomics, it was shown that the key transcripts related to aromatic hydrocarbon degradation in agricultural soil polluted with heavy chemical fertilisers and pesticides were highly expressed [29]. From there, better insights have been deciphered on the critical role of gene encoding for 4-hydroxyphenylpyruvate dioxygenase, glyoxalase resistance dioxygenase, metapyrocatechase, and ring-hydroxylating dioxygenases in the aromatic and hydrocarbon metabolisms, demonstrating the underlying mechanisms of microbiome function to overcome the stress contributed by excessive use of chemical inputs, hence directly improving soil fertility [50,51]. A recent work by Zafar et al. [52] extended the co-application of a PGPR strain in compost-mixed biochar to alleviate the lead (Pb) toxicity by enhancing potassium uptake and root growth in the crop. This study showed the efficacy of the 1-aminocyclopropane-1-carboxylase (ACC) deaminase producing PGPR (*Bacillus amyloliquefaciens*) in attenuating Pb in *Spinacia oleracea* L. (spinach) cultivated in Pb-contaminated soil. Through this knowledge, the PGPR strains expressing the pollutant degradation key genes could be inoculated in compost and biochar to treat polluted soil, subsequently improving the soil fertility without chemical inputs.

The excessive long-term application of chemical fertilisers, especially nitrogen-based fertilisers, may also result in soil acidification [53], which will eventually cause the increased solubility of toxic elements such as aluminium and manganese, which are naturally present in the soil in solid form. As the soil’s pH decreases, the aluminium will cause the plant roots to deteriorate, affecting the plant’s ability to absorb water and nutrients [46]. On the other hand, the toxic levels of manganese may disrupt the crop growth process, especially in the upper part of the plant, which results in stunted growth and poor productivity [54]. It is known that several bacterial taxa have specific mechanisms to survive and thrive in an acidic environment. Nevertheless, the application of these beneficial microbes in attenuating acidic agriculture soil due to excessive chemical fertiliser usage has not been adequately explored. Prior knowledge on the mechanisms of the bacterial community in an acidic environment has been previously documented, as shown in the study on the expression of genes in artificial acid mine drainage (AMD), which revealed an abundance of genes including *dsrAB*, *dsrD*, and *dsrL* encoding for dissimilatory sulphate reduction. In addition, the expression of other resistance genes such as genes encoding for iron metabolisms (*cyt572* and *cyt1*) and proton buffer molecule metabolisms (*pstACAB* for phosphate and *pdaD* for arginine) suggested the mechanisms of adaptation and response of bacteria to acid mine drainage [55]. The expressions of these key genes in extreme acidic, iron, and sulphur-rich AMD environments are also evident in other studies, which revealed the key iron-oxidising bacteria *Ferrovum* sp. [56] and the shift in the microbial community structure with the increment of autotrophic microorganisms such as *Leptospirillum* [57]. Both studies have illustrated the importance of the key biogeochemical processes, such as nitrogen, sulphur, and carbon cycling in soil with elevated acidity, which are regulated by the microbial community, providing a proof of concept for acidic agriculture soil amendment using microbes. These pioneering studies led to the idea that the beneficial environmental microbes or PGPR strains can be exploited further in mitigating the soil acidification problems brought by the excessive usage of chemical fertilisers. All in all, the extensive usage of compost, biochar, and PGPR strains as biofertilisers to replace chemical fertilisers and improve agricultural soil health may be achieved by exploiting the key genes in the microbiome, which can be acquired through the metatranscriptomics technique.

## 3. Disease-Suppressive Soils as Greener Alternatives against Biotic Stress

Crops are frequently affected by diseases caused primarily by pathogens such as insects, fungi, nematodes, viruses, and bacteria, resulting in significant declines in crop growth and productivity [58]. Generally, crop disease-preventive control involves the use of mechanical approaches (e.g., tillage or mowing), cultural control (crop rotation), and chemicals (e.g., insecticides, herbicides, and pesticides), whereby the latter has been widely adopted [59]. Unfortunately, the usage of chemicals in resolving this issue would adversely disturb the environment due to their chemical toxicants’ compositions, such as carbamates, ethylene dibromide, triazines, and thiabendazole. Making it worse, some pesticides are even notorious for lingering in the environment for long periods due to their high-water solubility and volatility characteristics [60]. In addition, a good chance for groundwater pollution occurrence is presumable, owing to the seeping of the highly soluble pesticides trickling down from the surface runoff into the soil. Unable to keep these risks of jeopardising agricultural ventures, biological control is therefore gratified as a safer alternative in crop disease management, concomitant with the promotion of SDGs 11 and 12. In this light, the evident role of certain microbiome species in suppressing biological disease in the soil, such as *Acidobacteria* in suppressing vanilla *Fusarium* wild disease (using 16S rDNA amplicon sequencing) [61] and *Streptomycetaceae* against root pathogenic fungus *Rhizoctonia solani* in sugar beet plant (using 16S rDNA microarray) [62], have been scientifically narrated. Up to this point, it is fathomable that the microbial community is a notable feature that capacitates the disease-suppressive soils in tackling pathogenic invasion, which indirectly promotes plant growth [63]. Nevertheless, the core mechanisms adapted by these beneficial microbes in suppressing crop disease is another challenge that must be addressed. With additional concerns for the complexity of the soil inhabiting a diverse taxon of microbes [64], the convention of metatranscriptomics has been imposed, as it offers efficient, high-throughput analyses to uncover not only the complex microbial community but also their functional roles.

The soil’s general disease suppression property is a natural and pre-existing characteristic of soil regulated by the competitive and antagonistic behaviours of the indigenous soil microbiomes [65]. In contrast, the ‘specific’ disease suppression property is driven by the behaviour of a specific microbial species, which is suppressive to a specific pathogen for a specific crop [66]. As previously discussed, the specific suppression ability of the soil can be induced by inoculating the key species which is known to suppress a specific disease into the soil. These beneficial microbes will then colonise the rhizosphere and compete with the soilborne pathogens through various mechanisms such as antibiosis, microbiostasis, and competition for substrate [67]. Despite that, the induction of specific disease suppression in soil requires understanding of the active microbial communities that contribute to the disease’s suppression. The need to identify the suppressive mechanisms and microbes which mediate these functions is critical to this biocontrol strategy. Therefore, integration with metatranscriptomics analysis is proposed to interpret the molecular interactions between the soil microbiota, pathogens, and host plants comprehensively. Understanding the critical genes and biochemical pathway that regulate a plant’s response to disease infections may become revolutionary in aiding crop management against biotic stresses.

A study conducted by Hayden et al. [20] provided new insights into the expression of microbial genes in the wheat rhizosphere soil that are suppressive towards *Rhizoctonia solani* AG8, a fungus that causes *Rhizoctonia* root rot and bare patch disease. Based on the metatranscriptomics analysis, the non-suppressive rhizosphere soil was dominated by superoxide dismutase and thioredoxin system genes that are encoded in defence against oxidative stresses. These genes are expressed mainly by *Pseudomonas* spp. and *Arthrobacter* spp., which survive *R. solani* infection due to their ability to produce protective proteins that can detoxify reactive oxygen species (ROS). The remarkable abilities of these species have also been recently discussed [68,69], implying the desirability of metatranscriptomics integration in the agricultural sector. On the other hand, the suppressive soils were dominated by *Stenotrophomonas* spp., *Buttiauxella* spp., *Pseudomonas* spp., and *Streptomyces* spp. due to their capabilities in expressing genes related to antimicrobial activity, which explains their prevalence in the suppressed soil. Other than the significant differentially expressed (DE) genes detected in suppressed soil, such as the polyketide cyclase gene and terpenoid biosynthesis gene (*dxs*), it was also reported that most of the DE genes are unannotated, indicating that there is a significant fraction of important genes that remain undiscoverable due to database limitation [70]. Even so, it is worth recognising the pioneered initiatives for transcriptional profile establishment so far, as this effort has paved the future direction of benefitting from the metatranscriptomics technology to accurately determine the underlying mechanisms of the molecular interplay of plant–microbe–pathogen interactions, which is vital for developing disease-suppressive soils.

Plant pathogens often co-exist in a complex and dynamic microbial community, complicating the investigation of the fundamental mechanisms pertaining to plant disease development. This is brought about by the colonisation of multiple pathogenic species and other microorganisms exhibiting negative, neutral, or beneficial interactions at the same time within that exact host plant [71,72]. The advancement of the high-throughput metatranscriptomics approach provides the unprecedented opportunity to characterise multiple pathogens, especially in complex diseases such as grapevine trunk diseases. It is reported that multiple grapevine trunk pathogens (GTPs) are often detected from a mature grapevine that has contracted this disease [73]. Through metatranscriptomics analysis, thousands of putative virulence expression factors of multiple pathogenic species were characterised, which could not be obtained by using single-species inoculations [32]. It was documented that *Eytypa lata* is the predominant species in the samples with GTP infections under vineyard conditions, followed by *Phaeomoniella chlamydospore* and *Diplodia seriata*, with putative virulence factors, carbohydrate-active enzymes, and transporters as the most expressed functions. This finding also revealed that the multiple pathogen species causing the same diseases also activate similar virulence functions.

A similar approach was explored to reveal the expression of differentially expressed genes (DEGs) for multiple stress responses in the *Arabidopsis* plant infected with the necrotrophic fungal pathogen *Botrytis cinera* (using an Affymetrix ATH1 whole-genome GeneChips microarray) [74]. It was shown that the upregulated genes due to *B. cinera* infection, which include *CCR2*, *CYP71A13*, *NATA1*, *SRG1*, and *EL13-2*, are responsive to phytohormones (salicylic acid, methyl-jasmonate, 1-aminoacyclopropane-1-carboxylate, and abscisic acid), indicating that hormones play a dominant role in the transcriptional programming of the *Arabidopsis* defence response towards *B. cinera* infection. Alongside that, the potential of the *RAP2.4* gene to serve in plant defence by regulating endogenous signal molecules and pathogen-derived effectors was also highlighted, whereas previous research had reported a similar elevation in gene expression under drought stress [75]. Thus, the information of the DEGs in response to plant infection makes it possible to understand the mechanisms underlying the plant defence system, which can subsequently be utilised to develop disease-suppressive soils and enable genetic modification of the crop with the desired defensive traits.

## 4. Development of Bioindicator in Environmental Biomonitoring

Environmentally sustainable agriculture entails not only crop productivity but also environmental long-term preservation and sustainability. It is widely reported that the quality of the water and soil ecosystems have been dramatically declining lately, attributable to contaminations from industrial agriculture effluent [76,77,78]. Generally, pollution management in the water ecosystem is more complicated than the soil ecosystem, as water bodies are commonly exposed to various anthropogenic pollutants, hence complicating the identification of pollution sources [79]. For decades, the physicochemical properties based on several parameters such as biochemical oxygen demand (BOD) and chemical oxygen demand (COD) have been used to monitor water pollution. Although this approach is considered well established, it cannot specifically identify the contamination source, which indirectly obstructs the correct assessment of early pollution signs and ultimately hinders the establishment of an effective environmental remediation plan. The use of analytical procedures such as ultra-high performance liquid chromatography coupled with mass spectrometry (UHPLC-MS/MS) in environmental pollutant detection [80,81] may help in providing data on the presence and concentrations of chemicals in the studied environment. However, they may not explain the specific mechanisms or the relationship between the biological systems and the pollutants, which must be understood to enable immediate proper measures to bioremediate the pollutant. Given the rising prevalence of environmental pollution due to unsustainable agricultural practices, it is essential to investigate the most appropriate biomonitoring alternatives to complement the existing environmental monitoring approach, thereby contributing to SDG 6 (Figure 1).

For this reason, the potential use of bioindicators as the specific pollutant indication tools has garnered extensive interest among researchers [79,82,83,84]. Over the recent years, numerous scientific investigations have been conducted to explore the microbiome profiles in various environments (Table 2), enabling the elucidation of the potential use of bioindicators to complement the existing pollution monitoring system. A bioindicator could be from any species or a group of species and other biomaterials or biochemicals that are sensitive to their surrounding environments and can be used to measure the ecosystem’s health. The previous common trend in water ecosystem biomonitoring used higher organisms such as fish [85,86], frogs or toads [87,88], and clams [89,90]. While these approaches are crucial for a deeper comprehension of these populations’ survivability in the affected areas, the routine monitoring and quantification of these species may not be feasible in detecting the specific contamination in a particular area. This is due to the unfavourable time taken for sampling, tedious morphological and species identification, external influence, and the slow and non-automatable sample throughput. Hence, the utilisation of lower organisms such as prokaryotes and their functional genes has emerged as an attractive candidate for bioindicator studies. Furthermore, the prokaryotes are particularly sensitive to environmental disruptions [91], making them an excellent indicator to detect the slightest changes in the environment explicitly.

The recent work of Zolkefli et al. [79] extended the idea of using bacteria as bioindicators to indicate the palm oil mill effluent (POME) final discharge pollution in river water. The authors demonstrated the potential use of *Alcaligenaceae* and *Chromatiaceae* as bioindicators based on the 16S rRNA gene amplicon sequencing approach. Moreover, the bacterial community shifts with changes in the dissolved organic matter in lake and inflow rivers were also reported using the 16S rRNA amplicon sequencing approach, which showed an increment of several groups of bacteria such as Nitrospirae, Chloroflexi, and Bacteroidetes [103]. More studies utilising 16S amplicon sequencing in developing microbial biomarkers were reported mainly for polluted agricultural soils [104,105]. Nevertheless, the DNA as the starting material in 16S amplicon sequencing could not validate the activity or viability of a species of interest. Hence, this issue has led researchers to employ the metatranscriptomics approach to fill the gap, giving them the upper hand in developing a more specific and reliable bioindicator [106,107].

Metabolically, microorganisms rely on chemicals scavenged from their environments to ensure their growth and survival against toxic chemicals. Genetically exploiting their crucial adaptive factors will foster the role of bioindicators to explicitly indicate contamination in a particular environment [108]. Therefore, it can be said that the bioindicator study was motivated by the assumption that the sensitivity of the indigenous microbiome to the changes in its environment is simultaneously reflected through the expression of functional genes. Unveiling the upregulated functional genes of the community through metatranscriptomics can be handy to detect and monitor the effect of pollution in a particular area. For instance, it is well known that the agricultural sector contributes to harmful methane emissions, especially in paddy fields [109]. With that reference, an interesting study was conducted on flooded rice fields to infer the underlying mechanisms of the anaerobic community regarding methane emission through the metatranscriptomics approach [99]. The incorporation of metatranscriptomics analysis in this study contributes to identifying the key genes to encode for polymer hydrolysis, propionate metabolism, and syntrophic acetate oxidation that regulate hazardous methane emission under varying weather conditions. In addition, it also revealed the community dominance of *Methanosarcinacea*, a soil methanogen commonly found to be involved in methane emission in paddy fields [110,111]. These research findings provide an essential clue to the potential bioindicator used to indicate excessive methane emission.

Previously, Zolti et al. [33] had reported an interesting proof-of-concept study on the gene expression profiles of the root microbiomes as in situ biosensors to reveal the environmental conditions encountered by both microorganisms and host plants, using tomato and lettuce as the studied samples. By elevating the soil’s salinity and pH through irrigation with wastewater, it was reported that these changes caused a significant increment of tubulin and the FK506 binding protein peptidylprolyl cis-trans isomerase’ genes (FKBP-type) in the host plant. The FKBP genes, which belong to the peptidylprolyl *cis/trans* isomerase superfamily, have been reported to regulate plant defence mechanisms against drought stress. Its expression was associated with a significant increase in the plant survival rate [112]. The root-associated microbial metatranscriptomics enrichment analysis showed the significantly overexpressed gene sub-systems, including NQR, tripartite ATP-independent periplasmic (TRAP) transporters, sodium-hydrogen antiporters, alginate metabolism genes, and *MSHA4*. The taxonomic profiling analysis also showed the increment of bacterial species under the Gammaproteobacteria phylum, commonly characterised by the ability to survive in wastewater [113,114]. Hence, by understanding the microbial taxonomic profiles and gene expression patterns of the crop under various stresses, we can utilise this information to develop a specific and accurate bioindicator to indicate the type of environmental stress. Determining the type of stress affecting the crop through in situ biosensors will also allow a more accurate treatment. However, it is apparent from the literature that there is still inadequate information regarding the principal mechanisms exerted by the biological indicators upon environmental changes in response to industrial agriculture activity. On this account, a more concise understanding, particularly of the transcriptional diversity of the microbes, is needed, whereby the application of metatranscriptomics technology is highly recommended for this purpose.

Amidst the competitive pooling of data and information on the important genes and beneficial microbes in the environment, there is an unending inquiry regarding the deliverability of this knowledge into building up the most appropriate monitoring tool for a particular pollution. Alongside the progression of the excellent meta-omics approaches, inclusive of metagenomics, metatranscriptomics, and metabolomics in exposing the changes to the microbiome community, genes, or relevant metabolites in the polluted environment, there are also unavoidable demands for a cost-effective, reliable, and rapid technique to assess pollution based on the targeted indicator. As one of the most worthwhile bioindication instruments, biosensors have been extensively researched and thus engineered to close these gaps.

Biosensors are classified based on their transduction principle (optical, electrochemical, and piezoelectric) or their recognition elements such as immunosensors (antibodies), aptasensors (aptamers), genosensors (nucleic acid), and enzymatic (enzymes) biosensors, whereby a majority of biosensors used in environmental monitoring are enzymatic biosensors, immunosensors, and aptasensors [115]. Relatively, metatranscriptomics data have been imperative in developing a sensitive biosensor in targeting the element of interest. For example, a microplate-based optical microbial biosensor was developed to monitor pesticide contamination in soil by detecting the level of methyl parathion (MP), a type of organophosphate (OP) compound acting as an insecticide. The biosensor was developed by exploiting the functional ability of *Sphingomonas* sp. in producing OP-degrading hydrolase (OPH) enzyme, which works by hydrolysing MP into p-nitrophenol (PNP), where it can be optically detectable by the microplate reader [116]. Extending this idea, a portable version of this technology has been proposed and modified into an absorbance-based biosensor by using a different type of organophosphate-degrading enzymes (recombinant methyl parathion hydrolase (MPH)) which is fused with glutathione-S-transferase (GST). The recombinant MPH-GST are covalently immobilised onto a chitosan-coated microplate and connected to a transducer system to detect MP, enabling simultaneous multiple sample detection on-site [117].

On top of that, DNA hybridisation biosensors or genosensors have also surged in popularity in environmental biomonitoring in recent years. The genosensor is made up of a sequence-specific and short synthetic oligonucleotide (also known as capture probe), which is fixed on the signal transducer surface, recognising the target DNA or RNA [118]. Technically, the sequence-specific probe is designed based on the identified marker genes, followed by immobilisation on the transducer’s surface. To apply this technology in environmental biomonitoring, the specific gene target must be identified before designing the genosensors [119]. For instance, by conducting metatranscriptomics analysis on natural marine bacteria and their functional marker genes, specific genosensors have been successfully developed to monitor oil pollution in seawater [96]. In addition, recent reports have adopted the application of genosensors in monitoring various major environmental pollution types such as algal blooms [120,121]. Meanwhile, aptasensors that use aptamers as the sensing element gained upheaved attention as an environmental pollution biosensor. Aptamers are single-stranded oligonucleotide sequences (DNA or RNA) that can fold into precise conformations and bind to corresponding ligands with great avidity and specificity, making them useful as recognition elements in a variety of assay systems [122]. The usage of electrochemical biosensors in monitoring pesticides and toxic metals has also been extensively discussed as of late [123], corresponding to digital technology advancement and prompting a more precise monitoring technique in assessing the state of nature with greater accuracy than ever before.

The most advanced biomonitoring tool to date would be the integration of satellite analysis through remote sensing, such as the Moderate Resolution Imaging Spectroradiometer (MODIS) sensor, mounted on Terra and Aqua satellite platforms, and the Medium Resolution Imaging Spectrometer (MERIS), deployed on the Envisat-1 environmental research satellite. These sensors were specifically designed to monitor the global ecosystem functions and changes in the land, atmosphere, ocean, and cryosphere through data collection in various indices with varying time scales [124]. The scientific community has been using remote sensing data acquired by these technologies to infer the current ecological status of a specific environment, including the ecological status of the water bodies. It is understood that elevated concentrations of chlorophyll a (Chl-a) from the proliferation of photosynthetic cells (phytoplankton) can reflect an increase in nutrient loads, where this pattern would indicate the eutrophication status of aquatic ecosystems [125]. Taking advantage of this factor, many studies have utilised the remote sensing strategy by using MODIS to acquire the data on Chl-a concentration levels to monitor the harmful algal bloom phenomenon in various geographical locations, such as in eutrophic water reservoirs [126], lakes [127], coastal waters [125,128,129], and rivers [130]. Satellite remote sensing is currently the only approach capable of providing global coverage and continuous measurements across space with relatively high spatial and temporal resolutions [131]. From this review’s perspective, even though there is still a great deal of work that can possibly be performed in this area, satellite remote sensing is indeed inspiring as a future promising biomonitoring tool applicable across diverse environmental monitoring purposes.

## 5. Summary and Future Outlooks

The incorporation of metatranscriptomics in elucidating the beneficial microbial transcriptome profiles can serve as a powerful approach to enhance environmental sustainability by reducing the unnecessary application of chemical fertilisers and pesticides. Furthermore, understanding the shift of gene expression towards changes in the environment also enables the efficient monitoring of the natural environments affected by anthropogenic activities. However, as discussed in this review, many research gaps need to be addressed to successfully implement the metatranscriptomics approach in industrial agriculture and the environment. Moreover, large fractions of protein sequences in databases are still considered hypothetical proteins with uncharacterised functions. The lack of characterised proteins in the databases contributes to another challenge in metatranscriptomics study, which can be addressed with complicated in silico analyses and, of course, through the isolation and characterisation study of the specific protein.

Nevertheless, a deeper understanding of the transcriptional landscape of a particular environment can be achieved by integrating with other meta-omics approaches, such as metagenomics, metabolomics, and metaproteomics, which would assist in advancing the agriculture field. Thus, there is a potential opportunity to develop plant–microbe associations using meta-omics methods to increase crop productivity. Notably, metatranscriptomics analysis has tremendously advanced and enriched the scientific field of agricultural research. Moreover, as sequencing technology is expected to become more affordable, accurate, and even portable, this development will contribute to productive industrial agriculture and a sustainable environment.

## Figures and Tables

**Table 1:** The applications of metatranscriptomics in elucidating the functional role of the microbiome to increase crop productivity, which contributes to SDGs 2, 11, and 12.

Applications	Sample	Aims	Findings	Reference
Improving soil fertility	Paddy soil	Provide novel insights into the diversity of the soil community responsible for reductive nitrogen transformation (RNT).	*Anaeromyxobacter* and *Geobacter* (Deltaproteobacteria) were key players in driving RNT in paddy soils. These genera harboured nitric oxide reductase gene (*nor*) and nitric oxide synthase gene (*nos*).	[28]
	Agricultural soil vs. organic soil	Elucidate the microbial community structure and function in organic soil vs. agricultural land, which has undergone prolonged usage of chemical fertilisers, pesticides, and herbicides.	The agricultural soils showed a high expression of genes related to aromatic metabolisms. For example, gene-encoding 2-nitropropane dioxygenase (*Blastomonas* sp.), hydroxylating dioxygenase (*Novosphingobium* sp.), and intradiol ring-cleavage dioxygenase (*Novosphingobium aromaticivorans*).	[29]
	Subtropical natural grassland soils	Elucidate the soil microbial structure and function in response to short-term seasonal variations (cold vs. warm season).	The prevalence of the predominant group (*Candidatus Koribacter, Mycobacterium, Bacillus, Rhodoplanes*) in both seasons supported the idea of using core microbiota in predicting community response to perturbation. Soil microbial taxa are more sensitive to environmental changes, while microbial functions are more stable throughout the year.	[30]
	Agriculture soil	Identify dominant species and major transcripts extracted from soil having a long history of chemical fertilisers and pesticide usage.	The major bacterial genera detected in this ecosystem were *Achromobacter, Pseudomonas, Bacillus,* and *Sphingobium*. High expression of transcripts encoding aromatic compound metabolisms (e.g., Catechol 1, 2-dioxygenase, Benzoate 1, 2-Intradiol ring-cleavage dioxygenase, and Gentisate 1,2-dioxygenase) was observed.	[31]
Crop stress management (disease)	Woody stems of grapevines	In planta profiling of putative virulence activities in the grapevine trunk disease (GTD) complex.	High abundance of CAZymes and transporters, followed by secondary metabolism, cytochrome P450s, and peroxidases in all pathogens. The pathogen species associated with the same disease activate similar virulence functions.	[32]
	Tomato and lettuce roots	Elucidate potential of microbial functional gene profiles and expression patterns as in vivo sensors of environmental stress affecting host and host-associated communities.	The plants irrigated with treated wastewater (to induce stress) showed significant enrichment of stress-associated root transcripts and proteins. For example, ubiquinone oxidoreductase gene (*nqr*) and tripartite ATP-independent periplasmic (TRAP) transporter correlated with the increase in pH and salt in the rhizosphere.	[33]
	Rhizosphere soil	Provide insights into the functional profiles of the rhizosphere microbiome in response to soilborne pathogens (*R. solani* AG8) and identify the essential genes that play a significant role in disease suppression.	The suppressive samples were dominated by *Stenotrophomonas* spp. and showed high expression of the polyketide cyclase gene and terpenoid biosynthesis gene (*dsx*). Polyketides and terpenoids are known as antimicrobial secondary metabolites.	[20]
	Leaves of healthy and infected basil plants	Develop a comprehensive pipeline to study the genes expressed in both the host plant (sweet basil) and its obligate downy mildew parasite (*Peronospora belbahrii*) without prior genomic information for either the plant host or the pathogen.	The genes encoding Arginine-any amino acid-Leucine-Arginine (RxLR) effectors were revealed as the candidate pathogen virulence genes and highly expressed during infection. The upregulated genes in the host plant (e.g., beta-glucanase and lipoxygenase) were proposed as candidate host defence genes that could be utilised in routine plant screening for disease.	[34]

**Table 2:** The application of metatranscriptomics in sustaining environmental health, contributing to SDGs 6 and 12.

Applications	Sample	Aims	Findings	Reference
Monitoring changes in the environment	River watersediments	Examine the connection between contaminant rates and transcription profiles of microbial genes and diversity	The genes encoding nitrate reduction, methanogenesis, and beta-oxidation were significantly upregulated in sediments with high polycyclic aromatic hydrocarbons, polychlorinated biphenyls, and metals. Most mRNA transcripts remained statistically consistent despite the strong contaminant gradient.	[92]
	River water sediments	Provide insights into microbial dynamics in freshwater hydrocarbon-rich environments	The key genes involved in energy metabolism were reported. Nitrogen metabolism: nitrate reductase gene (*napA*), nitrite reductase gene (*nirB*), and nitrite oxidase reductase gene (*norB*); sulphur metabolism: sulphite reductase gene (*dsrAB*); methane metabolism: methyl coenzyme M reductase A gene (*mcrA*) and heterodisulphide reductase gene (*hdrA*). Various hydrocarbon degradation pathways were reported, indicating microbial consortia’s ability to degrade a wide array of nitroalkanes and nitroaromatics.	[93]
	Mangrove’s microbiome	Analyse the local mangrove microbiome functionality and diversity	*Burkholderiaceae*, *Planctomycetaceae*, *Rhodobacteraceae*, and *Desulfobacteraceae* were the microbial core in the mangrove rhizosphere ecosystem. They were responsible for regulating the following: methane metabolisms (phosphoenolpyruvate carboxylase gene (*ppc*) and ribulose-phosphate 3-epimerase gene (*rpe*)), nitrogen metabolisms (nitrate reductase gene (*narG* and *narZ*)), and sulphur metabolisms (sulphite reductase (NADPH) hemoprotein beta-component gene (*cysI*) and phosphoadenosine phosphosulphate reductase gene (*cysH*)).	[94]
	River watersediments	Identify the potential use of novel clusters of gene biomarkers to monitor aquatic health with regard to increasing hydrocarbon exposure	Elevated polycyclic aromatic compound (PAC) concentrations caused significant shifts in the microbial metabolic processes. Significant overexpression of biodegradation genes such as alkane monooxygenase gene (*alkB*), benzoyl-CoA reductase gene (*badDEFG*), and benzoylsuccinyl-CoA dehydrogenase gene (*bbsBD*), indicating their potential as PAC biomarkers.	[95]
	Seawater	Identify the potential use of genes pool in genosensing to indicate oil contamination in seawater	The genes involved in alkane degradation (*alkB*) and aromatic degradation such as benzoate/toluate 1,2-dioxygenase components as well as dihydroxy-cyclohexadiene carboxylate dehydrogenase gene (*benABCD*) and benzoyl-CoA converting enzyme (*boxABC*) were upregulated in the oil-treated tank. Seasonal effect (e.g., phytoplankton senescence) was influenced the transcription profile.	[96]
	Coastal sediment	Outline the potential pathways involved in the production or degradation of nutrients regarding different levels of organic enrichment and metal contamination	Excess organic enrichment in coastal sediments resulted in the upregulation of genes involved in producing toxic ammonia, hydrogen sulphide, and nitrous oxide. Metal contamination in the coastal sediments did not significantly affect the gene profile.	[97]
Reducing negative impact on the environment	Cyanobacterial bloom (lake)	Provide an understanding of the roles and importance of cyanobacterial N_2_ fixation and phosphorous scavenging pathways during cyanobacterial blooms	The expression of genes involved in N_2_ fixation (*nifDKH*) and the phosphorous scavenging- phosphate-specific transport system genes (*pstS*, *pstB*, and *pstC*) were significantly upregulated during the bloom. The genes were majorly expressed by *Nostocales.*	[98]
	Flooded rice field soil	Clarify the impact of temperature on the structural and functional profiles of the anaerobic food web in rice field soil associated with methane production (mesophilic: 30 °C, thermophilic: 45 °C)	The mesophilic food web was characterised by progressive polymer breakdown that governed acetoclastic methanogenesis (*Methanosarcinaceae*) and syntrophic propionate oxidation (*Christensenellaceae*), with polymer hydrolysis becoming the rate-limiting phase. The thermophilic food web had two activity stages: polymer hydrolysis and syntrophic acetate oxidation (*Themoanaerobacteraceae*, and *Heliobacteriaceae*).	[99]
	Activated sludge	Identify the diversity, abundance, and expression of antibiotic resistance gene (ARG) hosts in activated sludge in wastewater treatment plants	The family *Burkholderiaceae* was reported to harbour 50 ARGs and was regarded as the most important ARG host in this study. The multi-drug resistant host was highlighted, with most of the bins annotated to Proteobacteria. The genus *Mycobacterium* harboured 14 ARGs, which conferred resistance to 10 different antibiotics.	[100]
	Seawater	Determine the mechanisms of natural oil-degrading bacteria in the presence of dispersants in the marine environment	The genera *Thalassolituus* (summer) and *Oleispira* (winter) were reported as the key players involved in oil degradation in the presence of dispersant. The addition of dispersant resulted in the enrichment of fatty acid degradation (*alkB*), an important metabolic function for oil degradation.	[101]
	Acid mine drainage	Provide insights into the role of iron-oxidising bacteria (*Ferrovum* sp.) in the bioremediation of arsenic	The *Ferrovum* sp. was metabolically active and harboured genes related to stress response, including an arsenic resistance gene cluster *(arsC*, *arsA*, and *arsD)* and general stress response genes (*yhdN*, and *nhaX*). The low level of genes involved in metal resistance (*cusABC*, and *czcABC*).	[56]
	Bioleaching	Explain underlying mechanisms adapted by microbial communities (acidophilic strains) in bioleaching	The microorganisms were able to adapt and survive in oligotrophic conditions (early stage) by enhancing the cell proliferation, catalytic activation, and binding action. Gene-encoding signal transduction, localization, and the transporter were highly expressed in the stressful late stage.	[102]

## References

[1] Godfray H.C.J., Crute I.R., Haddad L., Muir J.F., Nisbett N., Lawrence D., Pretty J., Robinson S., Toulmin C., Whiteley R. (2010). The future of the global food system. Philos. Trans. R. Soc. B Biol. Sci..

[2] Zhang L., Yan C., Guo Q., Zhang J., Ruiz-Menjivar J. (2018). The impact of agricultural chemical inputs on environment: Global evidence from informetrics analysis and visualization. Int. J. Low-Carbon Technol..

[3] Faure D., Bonin P., Duran R. (2015). Environmental microbiology as a mosaic of explored ecosystems and issues. Environ. Sci. Pollut. Res..

[4] Timmis K., de Vos W.M., Ramos J.L., Vlaeminck S.E., Prieto A., Danchin A., Verstraete W., de Lorenzo V., Lee S.Y., Brüssow H. (2017). The contribution of microbial biotechnology to sustainable development goals. Microb. Biotechnol..

[5] Cavicchioli R., Ripple W.J., Timmis K.N., Azam F., Bakken L.R., Baylis M., Behrenfeld M.J., Boetius A., Boyd P.W., Classen A.T. (2019). Scientists’ warning to humanity: Microorganisms and climate change. Nat. Rev. Microbiol..

[6] Shakya M., Lo C.-C., Chain P.S.G. (2019). Advances and challenges in metatranscriptomic analysis. Front. Genet..

[7] Vierheilig J., Savio D., Farnleitner A.H., Reischer G.H., Ley R.E., Mach R.L., Farnleitner A.H., Reischer G.H. (2015). Potential applications of next generation DNA sequencing of 16S rRNA gene amplicons in microbial water quality monitoring. Water Sci. Technol..

[8] Beale D.J., Karpe A.V., Ahmed W., Cook S., Morrison P.D., Staley C., Sadowsky M.J., Palombo E.A. (2017). A community multi-omics approach towards the assessment of surface water quality in an urban river system. Int. J. Environ. Res. Public Health.

[9] Mohd-Nor D., Ramli N., Sharuddin S.S., Hassan M.A., Mustapha N.A., Amran A., Sakai K., Shirai Y., Maeda T. (2018). *Alcaligenaceae* and *Chromatiaceae* as reliable bioindicators present in palm oil mill effluent final discharge treated by different biotreatment processes. Ecol. Indic..

[10] Zhang X., Qu Y., Ma Q., Zhang Z., Li D., Wang J., Shen W., Shen E., Zhou J. (2015). Illumina MiSeq Sequencing reveals diverse microbial communities of activated sludge systems stimulated by different aromatics for indigo biosynthesis from indole. PLoS ONE.

[11] Zuñiga C., Zaramela L., Zengler K. (2017). Elucidation of complexity and prediction of interactions in microbial communities. Microb. Biotechnol..

[12] Aguiar-Pulido V., Huang W., Suarez-ulloa V., Cickovski T., Mathee K., Narasimhan G. (2016). Approaches for microbiome analysis. Lib. Acad..

[13] Staley C., Gould T.J., Wang P., Phillips J., Cotner J.B., Sadowsky M.J. (2014). Core functional traits of bacterial communities in the Upper Mississippi River show limited variation in response to land cover. Front. Microbiol..

[14] Zaikova E., Goerlitz D.S., Tighe S.W., Wagner N.Y., Bai Y., Hall B.L., Bevilacqua J.G., Weng M.M., Samuels-Fair M.D., Johnson S.S. (2019). Antarctic relic microbial mat community revealed by metagenomics and metatranscriptomics. Front. Ecol. Evol..

[15] Gupta N., Vats S., Bhargava P. (2018). Sustainable Agriculture: Role of metagenomics and metabolomics in exploring the soil microbiota. Silico Approach for Sustainable Agriculture.

[16] Peimbert M., Alcaraz L.D. (2016). Field Guidelines for Genetic Experimental Designs in High-Throughput Sequencing.

[17] Azeem M., Soundari P.G., Ali A., Tahir M.I., Imran M., Bashir S., Irfan M., Li G., Zhu Y.G., Zhang Z. (2021). Soil metaphenomics: A step forward in metagenomics. Arch. Agron. Soil Sci..

[18] Mauchline T.H., Hayat R., Clark I.M., Hirsch P.R. (2018). Old meets new: Most probable number validation of metagenomic and metatranscriptomic datasets in soil. Lett. Appl. Microbiol..

[19] Schmidt J.E., Gaudin A.C.M. (2018). What is the agronomic potential of biofertilizers for maize? A meta-analysis. FEMS Microbiol. Ecol..

[20] Hayden H.L., Savin K.W., Wadeson J., Gupta V.V.S.R., Mele P.M. (2018). Comparative metatranscriptomics of wheat Rhizosphere microbiomes in disease suppressive and non-suppressive soils for *Rhizoctonia solani* AG8. Front. Microbiol..

[21] Rajarapu S.P., Shreve J.T., Bhide K.P., Thimmapuram J., Scharf M.E. (2015). Metatranscriptomic profiles of Eastern subterranean termites, *Reticulitermes flavipes* (Kollar) fed on second generation feedstocks. BMC Genom..

[22] Epelde L., Jauregi L., Urra J., Ibarretxe L., Romo J., Goikoetxea I., Garbisu C. (2018). Characterization of composted organic amendments for agricultural use. Front. Sustain. Food Syst..

[23] Pergola M., Persiani A., Palese A.M., Di Meo V., Pastore V., D’Adamo C., Celano G. (2018). Composting: The way for a sustainable agriculture. Appl. Soil Ecol..

[24] Wilson C., Zebarth B.J., Goyer C., Burton D.L. (2018). Effect of diverse compost products on soilborne diseases of potato. Compos. Sci. Util..

[25] Chan K.Y., Van Zwieten L., Meszaros I., Downie A., Joseph S. (2007). Agronomic values of greenwaste biochar as a soil amendment. Aust. J. Soil Res..

[26] Singh B., Singh B.P., Cowie A.L. (2010). Characterisation and evaluation of biochars for their application as a soil amendment. Aust. J. Soil Res..

[27] Uchimiya M., Lima I.M., Klasson K.T., Wartelle L.H. (2010). Contaminant immobilization and nutrient release by biochar soil amendment: Roles of natural organic matter. Chemosphere.

[28] Masuda Y., Itoh H., Shiratori Y., Isobe K., Otsuka S., Senoo K. (2017). Predominant but previously-overlooked prokaryotic drivers of reductive nitrogen transformation in paddy soils, revealed by metatranscriptomics. Microbes Environ..

[29] Sharma P.K., Sharma V., Sharma S., Bhatia G., Singh K., Sharma R. (2019). Comparative metatranscriptome analysis revealed broad response of microbial communities in two soil types, agriculture versus organic soil. J. Genet. Eng. Biotechnol..

[30] Barboza A.D.M., Pylro V.S., Jacques R.J.S., Gubiani P.I., de Quadros F.L.F., da Trindade J.K., Triplett E.W., Roesch L. (2018). Seasonal dynamics alter taxonomical and functional microbial profiles in Pampa biome soils under natural grasslands. PeerJ.

[31] Sharma R., Sharma P.K. (2018). Metatranscriptome sequencing and analysis of agriculture soil provided significant insights about the microbial community structure and function. Ecol. Genet. Genom..

[32] Morales-Cruz A., Allenbeck G., Figueroa-Balderas R., Ashworth V.E., Lawrence D.P., Travadon R., Smith R.J., Baumgartner K., Rolshausen P.E., Cantu D. (2018). Closed-reference metatranscriptomics enables in planta profiling of putative virulence activities in the grapevine trunk disease complex. Mol. Plant Pathol..

[33] Zolti A., Green S.J., Sela N., Hadar Y., Minz D. (2020). The microbiome as a biosensor: Functional profiles elucidate hidden stress in hosts. Microbiome.

[34] Guo L., Allen K.S., Deiulio G., Zhang Y., Madeiras A.M., Wick R.L., Ma L.J. (2016). A De Novo-assembly based data analysis pipeline for plant obligate parasite metatranscriptomic studies. Front. Plant Sci..

[35] Uzoh I.M., Babalola O.O. (2018). Rhizosphere biodiversity as a premise for application in bio-economy. Agric. Ecosyst. Environ..

[36] Gupta G., Parihar S.S., Ahirwar N.K., Snehi S.K., Singh V. (2015). Plant growth promoting rhizobacteria (PGPR): Current and future prospects for development of sustainable agriculture. J. Microb. Biochem. Technol..

[37] Trivedi P., Delgado-Baquerizo M., Trivedi C., Hamonts K., Anderson I.C., Singh B.K. (2017). Keystone microbial taxa regulate the invasion of a fungal pathogen in agro-ecosystems. Soil Biol. Biochem..

[38] Gómez-Godínez L.J., Fernandez-Valverde S.L., Martinez Romero J.C., Martínez-Romero E. (2019). Metatranscriptomics and nitrogen fixation from the rhizoplane of maize plantlets inoculated with a group of PGPRs. Syst. Appl. Microbiol..

[39] Lindström K., Mousavi S.A. (2020). Effectiveness of nitrogen fixation in rhizobia. Microb. Biotechnol..

[40] Nag P., Shriti S., Das S. (2020). Microbiological strategies for enhancing biological nitrogen fixation in nonlegumes. J. Appl. Microbiol..

[41] Malviya M.K., Li C.N., Solanki M.K., Singh R.K., Htun R., Singh P., Verma K.K., Yang L.T., Li Y.R. (2020). Comparative analysis of sugarcane root transcriptome in response to the plant growth-promoting *Burkholderia anthina* MYSP113. PLoS ONE.

[42] Arif M.S., Shahzad S.M., Riaz M., Yasmeen T., Shahzad T., Akhtar M.J., Bragazza L., Buttler A. (2017). Nitrogen-enriched compost application combined with plant growth-promoting rhizobacteria (PGPR) improves seed quality and nutrient use efficiency of sunflower. J. Plant Nutr. Soil Sci..

[43] Billah M., Khan M., Bano A., Nisa S., Hussain A., Dawar K.M., Munir A., Khan N. (2020). Rock phosphate-enriched compost in combination with rhizobacteria; a cost-effective source for better soil health and wheat (*Triticum aestivum*) productivity. Agronomy.

[44] Tran P., Tran P., Cao D. (2019). Effect of compost, NPK and plant promoting rhizobacteria (PGPR) on growth and yield of three vegetables cultivated on arenosols. Int. J. Environ. Agric. Res..

[45] Grgić D.K., Domanovac M.V., Domanovac T., Šabić M., Cvetnić M., Bulatović V.O. (2019). Influence of *Bacillus subtilis* and *Pseudomonas aeruginosa* BSW and clinoptilolite addition on the biowaste composting process. Arab. J. Sci. Eng..

[46] Xia H., Riaz M., Zhang M., Liu B., El-Desouki Z., Jiang C. (2020). Biochar increases nitrogen use efficiency of maize by relieving aluminum toxicity and improving soil quality in acidic soil. Ecotoxicol. Environ. Saf..

[47] Xing Y., Wang J., Shaheen S.M., Feng X., Chen Z., Zhang H., Rinklebe J. (2020). Mitigation of mercury accumulation in rice using rice hull-derived biochar as soil amendment: A field investigation. J. Hazard. Mater..

[48] Ren H., Huang B., Fernández-García V., Miesel J., Yan L., Lv C. (2020). Biochar and rhizobacteria amendments improve several soil properties and bacterial diversity. Microorganisms.

[49] Srivastava M., Srivastava A., Yadav A., Rawat V. (2019). Hydrocarbon Pollution and Its Effect on the Environment.

[50] Correa-García S., Rheault K., Tremblay J., Séguin A., Yergeau E. (2021). Soil characteristics constrain the response of microbial communities and associated hydrocarbon degradation genes during phytoremediation. Appl. Environ. Microbiol..

[51] Liang C., Huang Y., Wang Y., Ye Q., Zhang Z., Wang H. (2019). Distribution of bacterial polycyclic aromatic hydrocarbon (PAH) ring-hydroxylating dioxygenases genes in oilfield soils and mangrove sediments explored by gene-targeted metagenomics. Appl. Microbiol. Biotechnol..

[52] Zafar-ul-Hye M., Tahzeeb-ul-Hassan M., Abid M., Fahad S., Brtnicky M., Dokulilova T., Datta R., Danish S. (2020). Potential role of compost mixed biochar with rhizobacteria in mitigating lead toxicity in spinach. Sci. Rep..

[53] Wang C., Zheng M., Song W., Wen S., Wang B., Zhu C., Shen R. (2017). Impact of 25 years of inorganic fertilization on diazotrophic abundance and community structure in an acidic soil in southern China. Soil Biol. Biochem..

[54] Santos-Medellín C., Edwards J., Liechty Z., Nguyen B., Sundaresan V. (2017). Drought stress results in a compartment-specific restructuring of the rice root-associated microbiomes. mBio.

[55] Tan S., Liu J., Fang Y., Hedlund B.P., Lian Z.H., Huang L.Y., Li J.T., Huang L.N., Li W.J., Jiang H.C. (2019). Insights into ecological role of a new deltaproteobacterial order *Candidatus Acidulodesulfobacterales* by metagenomics and metatranscriptomics. ISME J..

[56] Plewniak F., Koechler S., Le Paslier D., Héry M., Bruneel O., Bertin P.N. (2020). In situ metabolic activities of uncultivated *Ferrovum* sp. CARN8 evidenced by metatranscriptomic analysis. Res. Microbiol..

[57] Sun W., Sun X., Li B., Xu R., Young L.Y., Dong Y., Zhang M., Kong T., Xiao E., Wang Q. (2020). Bacterial response to sharp geochemical gradients caused by acid mine drainage intrusion in a terrace: Relevance of C, N, and S cycling and metal resistance. Environ. Int..

[58] Savary S., Willocquet L., Pethybridge S.J., Esker P., McRoberts N., Nelson A. (2019). The global burden of pathogens and pests on major food crops. Nat. Ecol. Evol..

[59] Khan S.M., Ali S., Nawaz A., Bukhari S.A.H., Ejaz S., Ahmad S. (2019). Integrated pest and disease management for better agronomic crop production. Agronomic Crops Volume 2: Management Practices.

[60] Pereira V.J., Cunha J.P.A.R., Morais T.P., Ribeiro-Oliveira J., Morais J.B. (2016). Physical-chemical properties of pesticides: Concepts, applications, and interactions with the environment. Biosci. J..

[61] Xiong W., Li R., Ren Y., Liu C., Zhao Q., Wu H., Jousset A., Shen Q. (2017). Distinct roles for soil fungal and bacterial communities associated with the suppression of vanilla Fusarium wilt disease. Soil Biol. Biochem..

[62] van der Voort M., Kempenaar M., van Driel M., Raaijmakers J.M., Mendes R. (2016). Impact of soil heat on reassembly of bacterial communities in the rhizosphere microbiome and plant disease suppression. Ecol. Lett..

[63] Toyota K., Shirai S. (2018). Growing interest in microbiome research unraveling disease suppressive soils against plant pathogens. Microbes Environ..

[64] Carvalhais L.C., Dennis P.G., Tyson G.W., Schenk P.M. (2012). Application of metatranscriptomics to soil environments. J. Microbiol. Methods.

[65] Cook R.J. (2014). Plant Health Management: Pathogen Suppressive Soils. Encycl. Agric. Food Syst..

[66] Schlatter D., Kinkel L., Thomashow L., Weller D., Paulitz T. (2017). Disease suppressive soils: New insights from the soil microbiome. Phytopathology.

[67] De Corato U. (2020). Soil microbiota manipulation and its role in suppressing soil-borne plant pathogens in organic farming systems under the light of microbiome-assisted strategies. Chem. Biol. Technol. Agric..

[68] Cheng H.J., Sun Y.H., Chang H.W., Cui F.F., Xue H.J., Shen Y.B., Wang M., Luo J.M. (2020). Compatible solutes adaptive alterations in Arthrobacter simplex during exposure to ethanol, and the effect of trehalose on the stress resistance and biotransformation performance. Bioprocess Biosyst. Eng..

[69] Jayamohan N.S., Patil S.V., Kumudini B.S. (2020). Seed priming with *Pseudomonas putida* isolated from rhizosphere triggers innate resistance against Fusarium wilt in tomato through pathogenesis-related protein activation and phenylpropanoid pathway. Pedosphere.

[70] Ijaq J., Malik G., Kumar A., Das P.S., Meena N., Bethi N., Sundararajan V.S., Suravajhala P. (2019). A model to predict the function of hypothetical proteins through a nine-point classification scoring schema. BMC Bioinform..

[71] Peyraud R., Mbengue M., Barbacci A., Raffaele S. (2019). Intercellular cooperation in a fungal plant pathogen facilitates host colonization. Proc. Natl. Acad. Sci. USA.

[72] Abdullah A.S., Moffat C.S., Lopez-Ruiz F.J., Gibberd M.R., Hamblin J., Zerihun A. (2017). Host–multi-pathogen warfare: Pathogen interactions in co-infected plants. Front. Plant Sci..

[73] Niem J.M., Billones-Baaijens R., Stodart B., Savocchia S. (2020). Diversity profiling of grapevine microbial endosphere and antagonistic potential of endophytic pseudomonas against grapevine trunk diseases. Front. Microbiol..

[74] Sham A., Al-Ashram H., Whitley K., Iratni R., El-Tarabily K.A., AbuQamar S.F. (2019). Metatranscriptomic analysis of multiple environmental stresses identifies RAP2.4 gene associated with arabidopsis immunity to *Botrytis cinerea*. Sci. Rep..

[75] Yang S.U., Kim H., Kim R.J., Kim J., Suh M.C. (2020). AP2/DREB Transcription factor RAP2.4 activates cuticular wax biosynthesis in arabidopsis leaves under drought. Front. Plant Sci..

[76] Amić A., Tadić L. (2018). Analysis of basic physical-chemical parameters, nutrients and heavy metals content in surface water of small catchment area of Karašica and Vučica Rivers in Croatia. Environments.

[77] Che Nadzir N.S., Abdullah M.Z., Sulaiman F.R. (2019). Surface water quality in palm oil plantation. Malays. J. Fundam. Appl. Sci..

[78] Rheinheimer dos Santos D., Monteiro de Castro Lima J.A., Paranhos Rosa de Vargas J., Camotti Bastos M., Santanna dos Santos M.A., Mondamert L., Labanowski J. (2020). Pesticide bioaccumulation in epilithic biofilms as a biomarker of agricultural activities in a representative watershed. Environ. Monit. Assess..

[79] Zolkefli N., Ramli N., Shaidatul N., Mohamad-zainal L., Asyifah N., Zulkhairi M., Yuso M., Ali M., Maeda T. (2020). *Alcaligenaceae* and *Chromatiaceae* as pollution bacterial bioindicators in palm oil mill effluent (POME) final discharge polluted rivers. Ecol. Indic..

[80] Kadmi Y., Favier L., Harja M., Simion A.I., Rusu L., Wolbert D. (2015). A new strategy for pentachlorophenol monitoring in water samples using ultra-high performance liquid chromatography—Mass spectrometry tandem. Environ. Eng. Manag. J..

[81] Campos-Mañas M.C., Plaza-Bolaños P., Sánchez-Pérez J.A., Malato S., Agüera A. (2017). Fast determination of pesticides and other contaminants of emerging concern in treated wastewater using direct injection coupled to highly sensitive ultra-high performance liquid chromatography-tandem mass spectrometry. J. Chromatogr. A.

[82] Li C., Quan Q., Gan Y., Dong J., Fang J., Wang L., Liu J. (2020). Effects of heavy metals on microbial communities in sediments and establishment of bioindicators based on microbial taxa and function for environmental monitoring and management. Sci. Total Environ..

[83] Mantilla J.G., Gomes L., Cristancho M.A. (2017). The differential expression of *Chironomus* spp genes as useful tools in the search for pollution biomarkers in freshwater ecosystems. Brief. Funct. Genom..

[84] Teta R., Esposito G., Casazza M., Zappa C.J., Endreny T.A., Mangoni A., Costantino V., Lega M. (2019). Bioindicators as a tool in environmental impact assessment: *Cyanobacteria* as a sentinel of pollution. Int. J. Sustain. Dev. Plan..

[85] Saleh Y.S., Marie M.A.S. (2016). Use of *Arius thalassinus* fish in a pollution biomonitoring study, applying combined oxidative stress, hematology, biochemical and histopathological biomarkers: A baseline field study. Mar. Pollut. Bull..

[86] Sweidan A.H., El-Bendary N., Hegazy O.M., Hassanien A.E., Snasel V. (2015). Water pollution detection system based on fish gills as a biomarker. Procedia Comput. Sci..

[87] Cruz-Santiago O., Pérez-Maldonado I.N., González-Mille D.J., Espinosa-Reyes G., Martínez-Toledo Á., Ilizaliturri-Hernández C.A. (2021). Nondestructive biomarkers in giant toad (*Rhinella marina*) to assess the effect of complex mixture of pollutants in Coatzacoalcos River, Mexico. Environ. Toxicol. Pharmacol..

[88] Peluso J., Aronzon C.M., Acquaroni M., Pérez Coll C.S. (2020). Biomarkers of genotoxicity and health status of *Rhinella fernandezae* populations from the lower Paraná River Basin, Argentina. Ecol. Indic..

[89] Jiang W., Fang J., Gao Y., Du M., Fang J., Wang X., Li F., Lin F., Jiang Z. (2019). Biomarkers responses in Manila clam, Ruditapes philippinarum after single and combined exposure to mercury and benzo[a]pyrene. Comp. Biochem. Physiol. Part C Toxicol. Pharmacol..

[90] Solé M., Bonsignore M., Rivera-Ingraham G., Freitas R. (2018). Exploring alternative biomarkers of pesticide pollution in clams. Mar. Pollut. Bull..

[91] Cordier T., Lanzén A., Apothéloz-Perret-Gentil L., Stoeck T., Pawlowski J. (2019). Embracing environmental genomics and machine learning for routine biomonitoring. Trends Microbiol..

[92] Falk N., Reid T., Skoyles A., Grgicak-Mannion A., Drouillard K., Weisener C.G. (2019). Microbial metatranscriptomic investigations across contaminant gradients of the Detroit River. Sci. Total Environ..

[93] Reid T., Chaganti S.R., Droppo I.G., Weisener C.G. (2018). Novel insights into freshwater hydrocarbon-rich sediments using metatranscriptomics: Opening the black box. Water Res..

[94] Rampadarath S., Bandhoa K., Puchooa D., Jeewon R., Bal S. (2018). Metatranscriptomics analysis of mangroves habitats around Mauritius. World J. Microbiol. Biotechnol..

[95] Reid T., Droppo I.G., Weisener C.G. (2020). Tracking functional bacterial biomarkers in response to a gradient of contaminant exposure within a river continuum. Water Res..

[96] Knapik K., Bagi A., Krolicka A., Baussant T. (2020). Metatranscriptomic analysis of oil-exposed seawater bacterial communities archived by an environmental sample processor (ESP). Microorganisms.

[97] Birrer S.C., Dafforn K.A., Sun M.Y., Williams R.B.H., Potts J., Scanes P., Kelaher B.P., Simpson S.L., Kjelleberg S., Swarup S. (2019). Using meta-omics of contaminated sediments to monitor changes in pathways relevant to climate regulation. Environ. Microbiol..

[98] Lu J., Zhu B., Struewing I., Xu N., Duan S. (2019). Nitrogen–phosphorus-associated metabolic activities during the development of a cyanobacterial bloom revealed by metatranscriptomics. Sci. Rep..

[99] Peng J., Wegner C.E., Bei Q., Liu P., Liesack W. (2018). Metatranscriptomics reveals a differential temperature effect on the structural and functional organization of the anaerobic food web in rice field soil. Microbiome.

[100] Liu Z., Klümper U., Liu Y., Yang Y., Wei Q., Lin J.G., Gu J.D., Li M. (2019). Metagenomic and metatranscriptomic analyses reveal activity and hosts of antibiotic resistance genes in activated sludge. Environ. Int..

[101] Tremblay J., Fortin N., Elias M., Wasserscheid J., King T.L., Lee K., Greer C.W. (2019). Metagenomic and metatranscriptomic responses of natural oil degrading bacteria in the presence of dispersants. Environ. Microbiol..

[102] Ma L., Wang H., Wu J., Wang Y., Zhang D., Liu X. (2019). Metatranscriptomics reveals microbial adaptation and resistance to extreme environment coupling with bioleaching performance. Bioresour. Technol..

[103] Zhang L., Fang W., Li X., Gao G., Jiang J. (2020). Linking bacterial community shifts with changes in the dissolved organic matter pool in a eutrophic lake. Sci. Total Environ..

[104] Ventorino V., Pascale A., Adamo P., Rocco C., Fiorentino N., Mori M., Faraco V., Pepe O., Fagnano M. (2018). Comparative assessment of autochthonous bacterial and fungal communities and microbial biomarkers of polluted agricultural soils of the Terra dei Fuochi. Sci. Rep..

[105] Zhao Z.B., He J.Z., Geisen S., Han L.L., Wang J.T., Shen J.P., Wei W.X., Fang Y.T., Li P.P., Zhang L.M. (2019). Protist communities are more sensitive to nitrogen fertilization than other microorganisms in diverse agricultural soils. Microbiome.

[106] Delforno T.P., Macedo T.Z., Midoux C., Lacerda G.V., Rué O., Mariadassou M., Loux V., Varesche M.B.A., Bouchez T., Bize A. (2019). Comparative metatranscriptomic analysis of anaerobic digesters treating anionic surfactant contaminated wastewater. Sci. Total Environ..

[107] Trench-Fiol S., Fink P. (2020). Metatranscriptomics from a small aquatic system: Microeukaryotic community functions through the diurnal cycle. Front. Microbiol..

[108] Xu T., Perry N., Chuahan A., Sayler G., Ripp S. (2014). Microbial Biodegradation and Bioremediation.

[109] Chidthaisong A., Cha-un N., Rossopa B., Buddaboon C., Kunuthai C., Sriphirom P., Towprayoon S., Tokida T., Padre A.T., Minamikawa K. (2018). Evaluating the effects of alternate wetting and drying (AWD) on methane and nitrous oxide emissions from a paddy field in Thailand. Soil Sci. Plant Nutr..

[110] Li D., Ni H., Jiao S., Lu Y., Zhou J., Sun B., Liang Y. (2021). Coexistence patterns of soil methanogens are closely tied to methane generation and community assembly in rice paddies. Microbiome.

[111] Yuan J., Yuan Y., Zhu Y., Cao L. (2018). Effects of different fertilizers on methane emissions and methanogenic community structures in paddy rhizosphere soil. Sci. Total Environ..

[112] Yu Y., Li Y., Jia F., Zhao M., Li W., Sun Q., Li N., Li W., Meng Z. (2017). ZmFKBP20-1 improves the drought and salt tolerance of transformed Arabidopsis. J. Plant Biol..

[113] Paliaga P., Felja I., Budiša A., Ivanĉić I. (2019). The impact of a fish cannery wastewater discharge on the bacterial community structure and sanitary conditions of marine coastal sediments. Water.

[114] Unuofin J.O., Okoh A.I., Nwodo U.U. (2019). Recovery of laccase-producing gammaproteobacteria from wastewater. Biotechnol. Rep..

[115] Justino C.I.L., Duarte A.C., Rocha-Santos T.A.P. (2017). Recent progress in biosensors for environmental monitoring: A review. Sens. Switz..

[116] Mishra A., Kumar J., Melo J.S. (2017). An optical microplate biosensor for the detection of methyl parathion pesticide using a biohybrid of Sphingomonas sp. cells-silica nanoparticles. Biosens. Bioelectron..

[117] Senbua W., Mearnchu J., Wichitwechkarn J. (2020). Easy-to-use and reliable absorbance-based MPH-GST biosensor for the detection of methyl parathion pesticide. Biotechnol. Rep..

[118] Palchetti I., Bettazzi F., Baussant T. (2018). Nanotechnology and Biosensors.

[119] Hashemi Goradel N., Mirzaei H., Sahebkar A., Poursadeghiyan M., Masoudifar A., Malekshahi Z.V., Negahdari B. (2018). Biosensors for the detection of environmental and urban pollutions. J. Cell. Biochem..

[120] Orozco J., Villa E., Manes C.L., Medlin L.K., Guillebault D. (2016). Electrochemical RNA genosensors for toxic algal species: Enhancing selectivity and sensitivity. Talanta.

[121] Morais S.L., Barros P., Santos M., Delerue-Matos C., Gomes A.C., Fátima Barroso M. (2021). Electrochemical genosensor for the detection of *Alexandrium minutum* dinoflagellates. Talanta.

[122] Phopin K., Tantimongcolwat T. (2020). Pesticide Aptasensors—State of the Art and Perspectives. Sensors.

[123] Hara T.O., Singh B. (2021). Electrochemical biosensors for detection of pesticides and heavy metal toxicants in water: Recent trends and progress. ACS EST Water.

[124] Justice C.O., Townshend J.R.G., Vermote E.F., Masuoka E., Wolfe R.E., Saleous N., Roy D.P., Morisette J.T. (2002). An overview of MODIS Land data processing and product status. Remote Sens. Environ..

[125] Abbas M.M., Melesse A.M., Scinto L.J., Rehage J.S. (2019). Satellite estimation of chlorophyll-a using moderate resolution imaging spectroradiometer (MODIS) sensor in shallow coastal water bodies: Validation and improvement. Water.

[126] Germán A., Tauro C., Scavuzzo M.C., Ferral A. Detection of algal blooms in a eutrophic reservoir based on chlorophyll-a time series data from MODIS. Proceedings of the 2017 IEEE International Geoscience and Remote Sensing Symposium (IGARSS).

[127] Zhang Y., Ma R., Duan H., Loiselle S., Zhang M., Xu J. (2016). A novel MODIS algorithm to estimate chlorophyll a concentration in eutrophic turbid lakes. Ecol. Indic..

[128] Sun X., Wu M., Xing Q., Song X., Zhao D., Han Q., Zhang G. (2018). Spatio-temporal patterns of Ulva prolifera blooms and the corresponding influence on chlorophyll-a concentration in the Southern Yellow Sea, China. Sci. Total Environ..

[129] Kim H.C., Son S., Kim Y.H., Khim J.S., Nam J., Chang W.K., Lee J.H., Lee C.H., Ryu J. (2017). Remote sensing and water quality indicators in the Korean West coast: Spatio-temporal structures of MODIS-derived chlorophyll-a and total suspended solids. Mar. Pollut. Bull..

[130] Chen Z., Dou M., Xia R., Li G., Shen L. (2021). Spatiotemporal evolution of chlorophyll-a concentration from MODIS data inversion in the middle and lower reaches of the Hanjiang River, China. Authorea.

[131] Skidmore A.K., Pettorelli N., Coops G., Geller N., Hansen M., Lucas R. (2015). Agree on biodiversity metrics to track from space. Nature.

